# Participatory evaluation of the process of co-producing resources for the public on data science and artificial intelligence

**DOI:** 10.1186/s40900-023-00480-z

**Published:** 2023-08-14

**Authors:** Piotr Teodorowski, Kelly Gleason, Jonathan J. Gregory, Martha Martin, Reshma Punjabi, Suzanne Steer, Serdar Savasir, Pournamy Vema, Kabelo Murray, Helen Ward, Dorota Chapko

**Affiliations:** 1 University of Liverpool, Liverpool, UK; 2 Imperial Cancer Research UK Lead Nurse, Department of Surgery and Cancer, Imperial College London, London, UK; 3 Computational Oncology Group, Department of Surgery and Cancer, Faculty of Medicine, Imperial College London, London, UK; 4 School of Primary Care and Public Health, Imperial College London, London, UK; 5 Imperial College London, London, UK; 6 School of Public Health, Imperial College London, London, UK; 7 NIHR Applied Research Collaboration Northwest London, Imperial College London, London, UK; 8 National Institute for Health Research Imperial Biomedical Research Centre, London, UK

**Keywords:** Photovoice, Evaluation, Data science, Co-production, AI, Background

## Abstract

**Background:**

The growth of data science and artificial intelligence offers novel healthcare applications and research possibilities. Patients should be able to make informed choices about using healthcare. Therefore, they must be provided with lay information about new technology. A team consisting of academic researchers, health professionals, and public contributors collaboratively co-designed and co-developed the new resource offering that information. In this paper, we evaluate this novel approach to co-production.

**Methods:**

We used participatory evaluation to understand the co-production process. This consisted of creative approaches and reflexivity over three stages. Firstly, everyone had an opportunity to participate in three online training sessions. The first one focused on the aims of evaluation, the second on photovoice (that included practical training on using photos as metaphors), and the third on being reflective (recognising one’s biases and perspectives during analysis). During the second stage, using photovoice, everyone took photos that symbolised their experiences of being involved in the project. This included a session with a professional photographer. At the last stage, we met in person and, using data collected from photovoice, built the mandala as a representation of a joint experience of the project. This stage was supported by professional artists who summarised the mandala in the illustration.

**Results:**

The mandala is the artistic presentation of the findings from the evaluation. It is a shared journey between everyone involved. We divided it into six related layers. Starting from inside layers present the following experiences (1) public contributors had space to build confidence in a new topic, (2) relationships between individuals and within the project, (3) working remotely during the COVID-19 pandemic, (4) motivation that influenced people to become involved in this particular piece of work, (5) requirements that co-production needs to be inclusive and accessible to everyone, (6) expectations towards data science and artificial intelligence that researchers should follow to establish public support.

**Conclusions:**

The participatory evaluation suggests that co-production around data science and artificial intelligence can be a meaningful process that is co-owned by everyone involved.

**Supplementary Information:**

The online version contains supplementary material available at 10.1186/s40900-023-00480-z.

## Abstract (plain language summary)

Modern technology offers new treatment options for patients and novel avenues of research. However, there is limited available information in easily understandable language for the public explaining how technology relates to them and could influence their healthcare. The researchers, healthcare professionals and public members worked together collaboratively to address this problem by creating new materials for the public. Our paper explores that project through creative methods. Firstly, everyone involved was offered an opportunity to attend training sessions. Then, people took photos and described them to illustrate to others what is their experience of working together. Finally, we all met to use included photos as building blocks to present a shared experience in the project. Afterwards, the professional artist included it as one circular illustration with six interlinked layers. These layers present everyone’s experiences (from inside) (1) is about the opportunity to build confidence in a new topic, (2) relationships with others, (3) working remotely during the pandemic, (4) motivation that influenced people to become involved in this particular piece of work, (5) expectation that the project needs be inclusive and accessible, (6) ethical principles that researchers using new technology should follow. We showed that it is possible for researchers, healthcare professionals and members of the public to feel joint ownership of the project and that working together can be meaningful to everyone.

**Supplementary Information:**

The online version contains supplementary material available at 10.1186/s40900-023-00480-z.

New technology underpinned by data science and artificial intelligence (AI) offers unique solutions for healthcare services and research. Data science and AI have an increasing influence on all our lives. The public is mostly supportive of reusing their data for health research but remains concerned about how this is being conducted [1–3]. They are often familiar with artificial intelligence but mostly thanks to examples from outside the healthcare settings [4]. In a data-rich healthcare system, the public should have access to information which explains how these technologies use data and AI. Access to this information can enable patients to ask questions about healthcare technologies and make informed decisions about using them. In some instances, patients might ask healthcare professionals about data science and AI. However, healthcare professionals might not have the time, knowledge or feel confident explaining artificial intelligence, or why data is collected and how reused if they are not directly responsible for it [5].

To help to address this emerging issue, our research team at Imperial College London, consisting of academic researchers and health professionals alongside 30 patients and members of the public (later referred to as public contributors) worked together on a public involvement project to co-design and co-develop an information resource in order to learn more about data science and AI in a healthcare setting. Public contributors were recruited from the patient involvement group for cancer research at Imperial College London, an Equality, Diversity & Inclusion group at Guy's & St Thomas's Hospital and an information governance committee at Patient Experience Research Centre. The breakdown was 60% women, 40% men, ages range 18–78, ethnicity White British, Eastern European, African, Caribbean, Asian. The project team (KG, JJG and MM) ensured that the involvement is accessible to public contributors by supporting them throughout the project. Involvement requires ongoing support, practical and monetary investments to be conducted meaningfully[6]. In this project, for example, public contributors who preferred hard-paper copies would receive one and could have sent the comments back on paper. The postage was reimbursed. This feedback was later incorporated by the project team to a digital version. Public contributors were recruited from several different patient and public involvement groups across medical specialities. The aim was to contribute resources that could increase digital health literacy to aid both health professionals and the public in making more informed and personal decisions in this increasingly digital world.

In our work, we perceive public involvement as a work "being carried out 'with' or 'by' members of the public rather than 'to', 'about' or 'for' them” [7]. We followed the UK Standards for Public Involvement to ensure the quality of involvement in our project [8]. In line with these principles, four public contributors are also co-authors of this paper.

Providing introductory information for public contributors around data science is not new; e.g. Teng and colleagues [9] provided members of the public with a booklet written by researchers on what linked data sets are and contentious issues around their use to provide a base for the discussion. Our project is novel because it aimed to co-develop materials with public contributors around data science and artificial intelligence.

Discussing data science and artificial intelligence with public contributors can be challenging because of the need to present appropriate information using lay language [10–13], as jargon and specialist terms can marginalise anyone unfamiliar with that vocabulary [14]. Public contributors might be apprehensive about joining involvement groups that focus on what they perceive as technical topics [15]. Involving those with a better understanding of the topic could overcome this barrier but such groups could have different views than a wider population [16]. Some researchers report that public contributors may not be interested in discussing data science [17] and can be more comfortable with involvement in qualitative rather than quantitative research (thanks to their lived experiences) [18]. To facilitate public involvement in complex topics additional training will be required for public contributors and researchers [19].

This paper reports on an evaluation of this novel approach to the development of materials on data science and artificial intelligence. We aimed to understand the co-production process through the following specific objectives:

1. To describe the experiences of working together on the project.
2. To explore how the process supports the personal development of public contributors.
3. To gain insights into the co-production process.

## Methodology

**Fig. 1:**
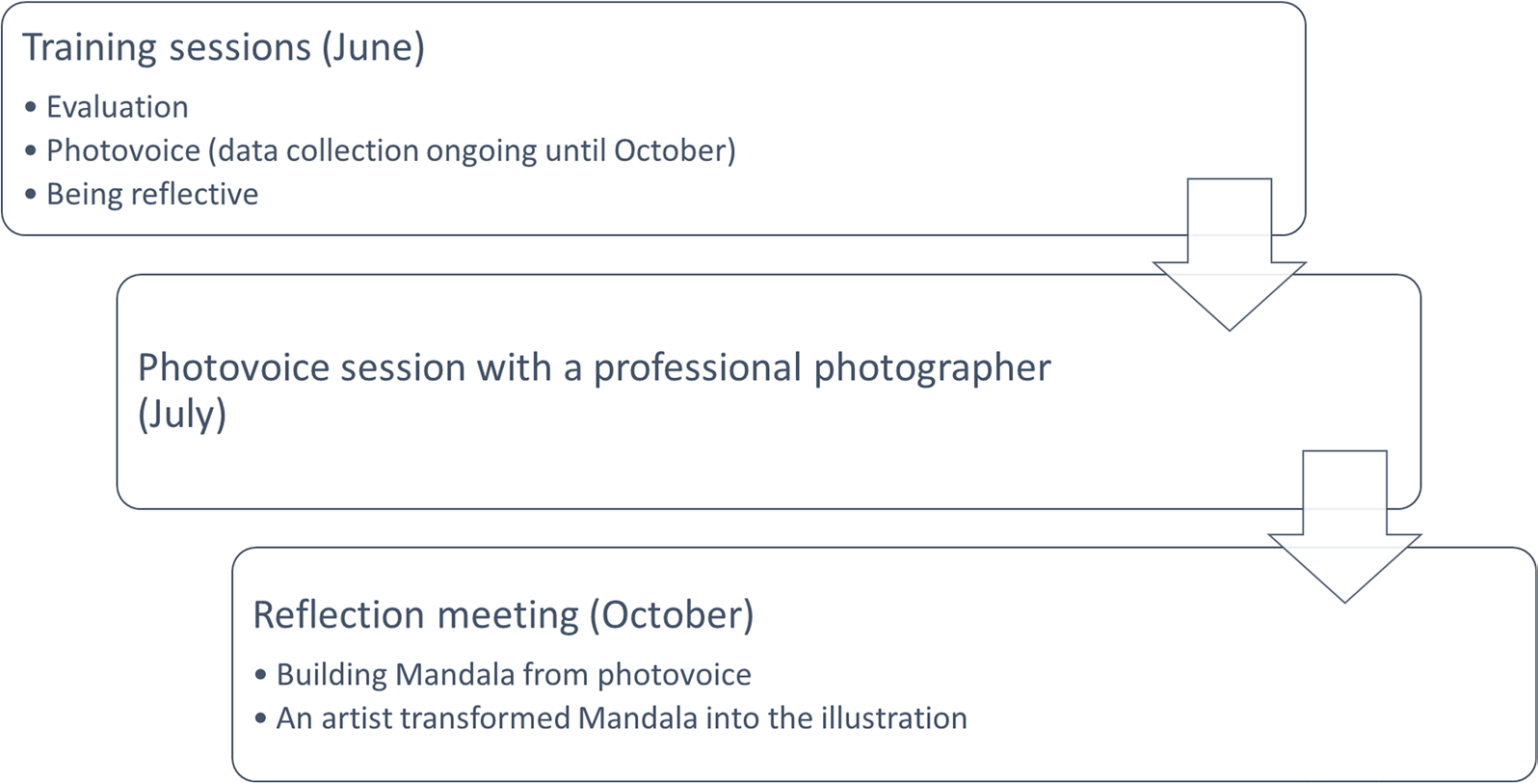
Summary of evaluation process

We adopted a participatory evaluation approach where public contributors, researchers and evaluators were directly involved in planning, conducting and analysing the evaluation [20]. It has many similarities with public involvement as it rebalances the power between those involved. This approach was jointly agreed between everyone involved. Participatory evaluation has been successfully used to evaluate public involvement [21]. We checked our reporting of public involvement in this evaluation using the GRIPP2 short form [22] (see Additional file 1). Throughout the evaluation process, we used creative methods [23] to make involvement more easily understood and appreciated by public contributors [24–26]. In the previous research, Tierney and colleagues [24] produced artwork to present public contributors' views and found it to be a more accessible way to disseminate research. In this section, we present the participatory evaluation process as summarised in Fig. 1.

The evaluation consisted of three stages. Firstly, PT delivered three training sessions on evaluation aims, photovoice and reflexivity. Secondly, utilizing photovoice public contributors captured their experience by choosing photos and using them to reflect and describe their experiences. Thirdly, using collected photos, paintings, and poetry, we built a joint experience of involvement as a ‘mandala’ turned a geometric configuration of symbols (or photos in our case) that represent the journey from the inner core to outside through different layers. Mandala was turned into a professional illustration. The evaluation process was embedded into already ongoing project sessions with evaluators joining existing meetings.

The evaluation was in a hybrid model, with activities taking place remotely and in person. This follows recent research showing that many public contributors prefer a mix of in-person and remote involvement [27]. All online sessions and the theoretical part of the in-person meeting with the photographer were recorded for those who could not attend. Meaningful remote public involvement is possible [28, 29]. However, it might require additional support for those who feel less comfortable with technology [27]; we provided additional instructions, for example, how to upload or comment on photos on the Padlet (an online notice board). We also offered one-to-one technical support for public contributors. This included assisting a public contributor to be one of the panellists during the Tweet Chat.

### Stage 1: training sessions

The first training focused on the aim of the evaluation. During the second one, everyone has received training around photovoice. We discussed ethics, photovoice aims, brief instructions on taking photos and reassured everyone that they could use phone cameras as no professional equipment is needed. In contrast to some other photovoice projects [30, 31] we did not provide disposable cameras as people felt that they would have access to one on their phone or tablet. When introducing the concept, everyone looked at sample photos and was asked to choose one representing their experience and share it with others during the discussion. This exercise aimed to help people reflect on how to link photos to their experiences. This is followed by another piece of training on reflexivity. Being reflective has become recognised as an important feature in research [32, 33]. Understanding our biases helps us to see what we bring to the discussion. Reflection is an ongoing process that starts even before research begins and is never final and complete. As public contributors reflected on their experiences through photovoice and were later involved in the analysis, it was crucial for them to reflect on their biases.

### Stage 2: photovoice

Photovoice, developed by Wang and Burris [34], is a participatory research method that utilises photos taken by participants. Photovoice is embedded in literature around feminism, community-based approaches and education for critical consciousness [34, 35]. Photos (taken and selected by people) accompanied by stories behind their meaning allow their authors to reflect more critically and present their experiences [36, 37]. Joining photos and words offers a more reflective space than when these modalities are presented in isolation. Authors of photos become more empowered than traditional study participants as they can decide the focus of the discussion rather than the researchers. It fosters trust with researchers and creates a sense of ownership among participants [38]. Photovoice is a popular method to empower seldom-heard groups [35, 39, 40] and has been previously used to involve communities around health issues [31].

Photovoice is a flexible method, and it can be successfully adapted to suit different contexts, groups and projects [41]. Following the principles of participatory evaluation, researchers, evaluation facilitators and public contributors were invited to participate in photovoice. Hence, we refer to everyone as participants. Everyone's voice was equal, allowing us to break power disbalances between evaluators, professionals and public contributors.

Photovoice does not require professional photographing skills. However, during discussions with public contributors, we recognised that some do not feel comfortable taking good-quality photos themselves. Therefore, we organised an in-person session with a professional photographer who provided tips about taking photos and then people had an opportunity to practise with the photographer on a one-to-one basis. This allowed people to build confidence when positively appraised by a professional photographer [42]. The session took place in a local charity centre in central London, thus ensuring enough room for everyone to move around and take photos. They were encouraged to bring some props to take a photo of that to express their experience of being involved in the project. One public contributor could not take photos themselves due to tremors in their hands so one of evaluators support them. The public contributor created a scene from their props, and the evaluator took a photo of it. Then, we discussed all photos taken during the session as a group. Although this was not compulsory, many of them were later selected for photovoice. The session's main aim was for people to improve their confidence when taking photos.

Everyone was invited to take photos and upload them online on Padlet. We kept it as a living document where photos and descriptions were available to see and comment on as the project progressed. This was similar to Fedorowicz and colleagues [43] use of Facebook for public involvement to allow more exchanges among public contributors. Many people used the opportunity to comment on each other's photos and encouraged others by praising their reflections or/and photos.

### Stage 3: reflection meeting and dissemination

During three months period, over 60 photos (alongside paintings and poetry) were uploaded online. Photos could be reviewed individually or in a group. We organised a reflection event to look at all taken photos and choose these representing common experiences. This was achieved by building a mandala. It offers an opportunity for people to reflect and produce new data [44]. Photovoice offered individuals perspectives on the project. The mandala assisted in organising the data from the photovoice. We used it to put together everyone's experiences to represent a shared journey. This was a joint analysis during which we identified connections and relationships between different aspects of the process and built consensus around a shared experience. Mandala was built by public contributors, researchers and evaluators. We brought the graphic designer who turned our mandala into an illustration. The draft was distributed to everyone involved (not only those attending the in-person meeting) for feedback. Six people used this opportunity meaning that eleven people gave their feedback on the process, and the illustration was updated accordingly.

Photovoice is an innovative and approachable way to reach external audiences such as other public contributors, researchers or policymakers [30, 35]. Because of the ongoing Covid-19 pandemic, we decided to go for online dissemination. We utilised Tweet Chat as this offered an open space to discuss the project with registered Twitter users (and was available to read by anyone). Tweet Chat has been utilised successfully by other researchers [24]. During our Tweet Chat using #JourneyIntoAI [45], we shared the draft Mandala and the selection of photos and invited a public involvement expert and an experienced public contributor not related to our project to join us in the online conversation.

### Ethical considerations

The Imperial Research Governance and Integrity Team (RGIT) was consulted about the proposed activities prior to starting. As this study is classified as evaluation and public involvement, all involved in the project are considered as collaborators not sources of data, and therefore no ethical approval is required [46]. However, we recognise the ethical considerations of using photovoice [42, 47]. Explicit consent for photo reuse was provided. Everyone's ownership of their photos remains in place, and we recognise everyone's contribution, for example, by co-authoring this paper with public contributors (everyone who took part in photovoice and attend one of face-to-face sessions could participate; public contributors decided themselves to which paper section they wanted to contribute). To avoid additional consent, we asked people not to take photos of people not involved in the project. We did not expect that sharing of included photos could impact anyone negatively. However, one public contributor requested assurance that their photos would not be identifiable. As meetings included working as a group we established ground rules to ensure everyone felt welcome and respected.

## Results

**Fig. 2:**
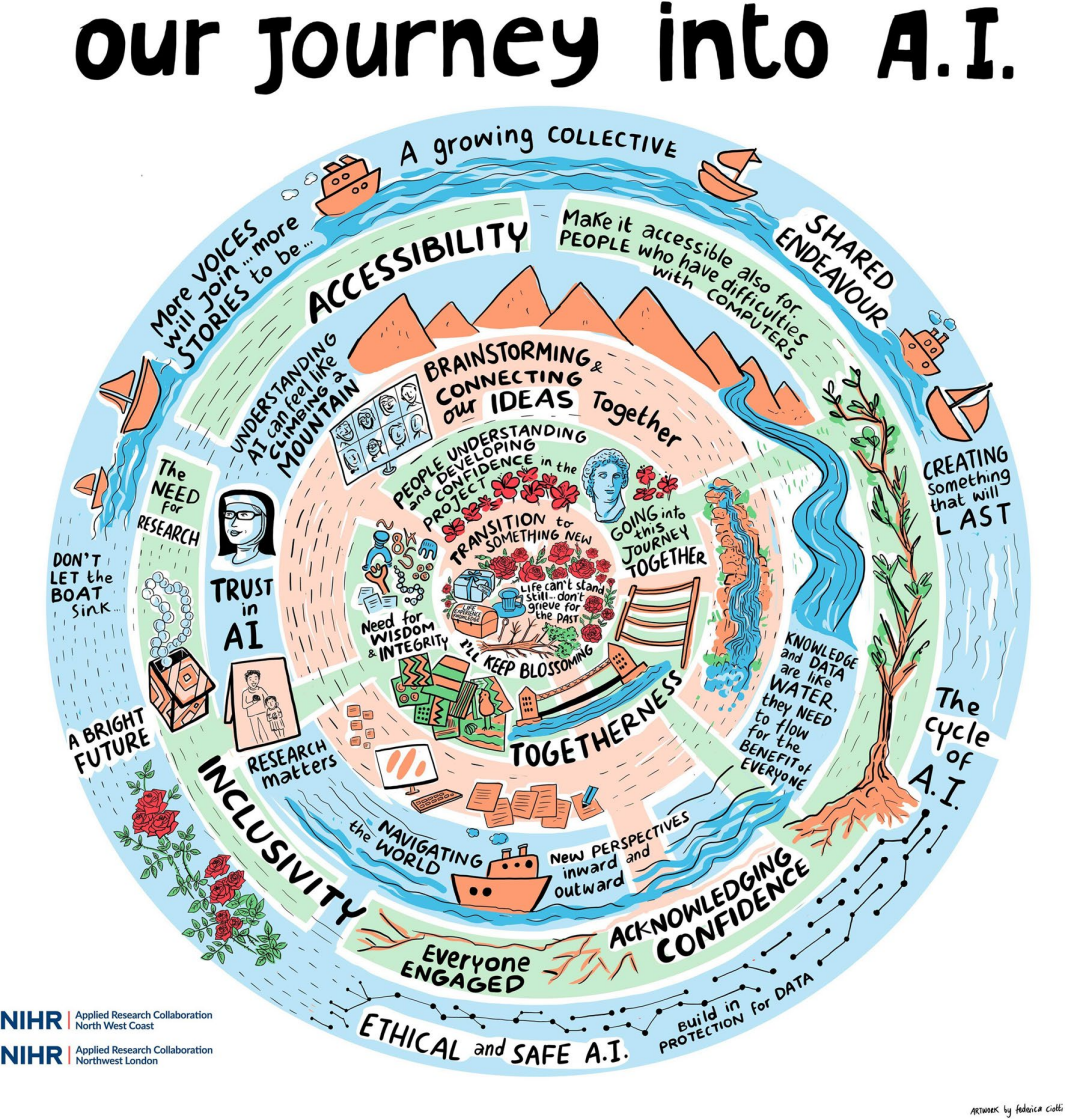
Mandala

We constructed the mandala (see Fig. 2) to illustrate everyone's experience of being involved in the project. These experiences are spread along six interconnected layers embodied as a connected journey. The chosen title, "Our Journey into A.I.", reflects the feeling of ownership among participants. The water and boats are present throughout as these "*express the sense of adventure, sailing into new experiences as a team*" (Public contributor 4). This emphasises that multiple bidirectional communication channels exist between all six layers of the mandala. This section discusses each layer of the mandala, starting from the inner core and moving towards to most outwards. We also use original commentaries of the photos collected during photovoice and used during the reflection meeting to provide more detailed descriptions. Each layer consists of related stories chosen by participants, but we would not consider them equal to themes (like could be found in thematic analysis). Participants recognised that issues discussed in inside layers could not have happened without those in more outward ones.

### Layer 1

The project offered public contributors an opportunity to develop as individuals, and learn new things, for example, improved knowledge of data science and artificial intelligence (especially as the majority of public contributors had limited understanding of the topic) and building confidence to be involved in other future projects. Flowers in the centre symbolise growth and transition into something new (often unexpected) in how participants developed but also hope that they will keep blossoming afterwards personally and as public contributors. However, that development was a process as explained by the public contributor:

"Two pictures of the red flower show my increased confidence, understanding and knowledge of data science and artificial intelligence. I am showing my new interpersonal and communication skills by displaying more confidence" (Public Contributor 2).

These are accompanied by four things: a fallen tree, the chest, the cup and the wrapped gift that summarise participants' experiences on a personal level and worth:

"The drawing of this fallen tree in the park, shows a tree having unfruitful branches while other branches are alive and blossoming. Being part of this project tells me I am still able to contribute hopefully something of value. The chest speaks for itself as the project enables me to use life's experience and knowledge. The cup may seem odd but I am often told I am doing too much. I should rest more. I can feel like a precious porcelain cup that should be on display rather than still being used for tea. And the wrapped gift is an expression of my gratitude in being asked to participate in this project" (Public contributor 4).

### Layer 2

This layer elicits participants' interactions and connections with aspects of data science, artificial intelligence, and other participants. The slogans “togetherness” and “going to into this journey together” recognise that this was a team work and the connection with other participants is described as a "bridge near the Thames":

"I chose this photo because of the connectivity that we all had. Despite being miles away from each other we were all still connected via Teams, emails etc. Just like a bridge connecting two places allowing a passage route. "(Public contributor 6).

Their joint experiences and work were explained with the references to human history as described by one of the public contributors using analogy to Alexander the Great through. This poem explains how they perceived project aims:

"We have moved forward since the days of Achilles and Alexander.

But

Our scientific community today is helping us wage war using the help of

Artificial intelligence.

Astonishingly, for the same needs and motives-

*Enhancement of human life*." (Public contributor 1).

The connections between the project topics, what participants found were unexpected as elicited by these two descriptions of photos:

"I love seeing patterns. Mathematical ones, those in nature [some tallying with Fibonacci numbers] and those designed by us humans e.g. the Kente cloth woven in Ghana. And so with Machine Learning. What patterns will be discovered with this data?" (Public contributor 4).

"We live in a world bombarded with data. As with ‘Alice in Wonderland’ one can desire at times to be free from data. Throw it away! I have become more aware of how data can be misused through ignorance but also by deliberately being messaged. There is need for integrity and WISDOM [hence the Pearls]. And the pennies? they represent the joy of those moments when in the confusion THE PENNY DROPS." (Public contributor 4).

### Layer 3

That connection with other participants (as being one team) continues in this layer, although here it is mostly related to digital technology that takes a centre place here. The photo of a computer screen with faces symbolises working online as the project took place remotely. This is how participants saw each other during meetings. Participants recognised that sometimes it was challenging staying active through full two-hour meetings in the evening. More than one photo (or screenshot) of zoom were included. These were captured under different circumstances to record various emotional stages of the co-production:

“*The breakthrough. …but we were never too far away from a good laugh*.” (Public contributor 7, describing the photo with zoom participants smiling).

“The blood sugar drop. It wasn't always easy to keep going through a two hour meeting from 5 to 7 pm…” (Public contributor 7, describing the photo with zoom participants looking tired).

The computer with keyboard and papers refer to their experience of note taking and providing feedback to draft materials.

"This picture represents me having to read through the different chapters for the project. I was intrigued and interested to learn more about the case studies and the information from the different chapters. It makes me feel like I am making a difference with my opinions and input." (Public contributor 3).

### Layer 4

This layer underpins why people got involved in this project in the first place. It includes an old photo of one of the researchers with their aunt, who was diagnosed with a mental illness. This is how they described their gratitude towards everyone's involvement:

"She was diagnosed late in life with bipolar disease. I remember how she struggled and I wonder what her life might have been like if we had the means to diagnose and treat bipolar disease back then. Research matters if we are going to improve how illness is diagnosed and managed. We need everyday people to work with researchers to develop new treatments and new ways of managing disease. That is why it is important that we are here together learning about an emerging field that will very likely revolutionise healthcare. That is why I am grateful, that for all the ways you could spend your time, you choose to spend it here, with us." (Researcher 1).

However, participants also pointed out that they might not have been so interested in it at the beginning, as explained by this public contributor:

"I chose this photo because despite it being a topic I am not particularly interested in, like the waterfall I was able to immerse myself in the calm and chaos that the project presented. I had found myself brainstorming so many ideas/thoughts that it felt chaotic at times but simultaneously I felt calm in being able to build upon the opinions and suggestions everyone else had as well." (Public contributor 6).

This process of involvement had a positive outcome for participants:

"[artificial intelligence] *is like rocky mountain to climb, but worth it*." (Public contributor 3).

This layer also includes a recognition of the role of researchers who supported public contributors. The woman in the photo is one of the researchers working on the project (KG). Public contributors wanted to point out that their experience (and outcomes included in previous layers) would not have been possible without the researchers’ help and support.

### Layer 5

This layer explores principles around public involvement and their expectations towards what should take place to ensure that the co-production process is inclusive and accessible to everyone. Everyone needs to be engaged, but this also means public contributors have to be empowered to do it by building confidence. Remote working has challenges, and participants recognised that involvement must be accessible for people who have computer difficulties. That support needs to be ongoing, and people feel comfortable requesting it. During the reflection meeting, public contributors said that they felt these requirements were met. Ensuring the supporting environment led to improved confidence and pride as the project was progressing, as shown in the photo titled the “Japanese Puzzle Box and Pearls”:

"How do you open the box? It is not easy—it can take hours to fathom how. Grappling with AI, particularly machine learning, is not easy and yet in gaining some understanding it brings a sense of discovering pearls of knowledge" (Public contributor 4).

### Layer 6

The most outmost layer shows what the participants perceived as underlying principles of data and artificial intelligence usage for research and healthcare services. They were: ethical and safe artificial intelligence, creating something that will last (as this should not be a one-off event or improvement), change should be a shared endeavour, and if more people are involved then more stories will be included (thus more meaningful change will take place). As this public contributor expressed in this poem around their red roses, when done in an appropriate way data science and artificial intelligence can make a real-life difference:

"The red rose tree, is about 60 years old.

The yellow rose tree, is about 40 years old.

They enhance my life in joy and serenity with their beauty.

They demand careful care to do this, in feeding and pruning and watering. Then the roses flourish and only bring happiness.

This reminds me, of the artificial intelligence, I am studying at the moment.

Like my roses, when artificial intelligence is given careful care, pruning the parts that could endanger, feeding it the right ideas, it will enhance health and life, in joy and serenity. "(Public contributor 1).

These principles were also applicable to what participants expected from this project. Without them being met, they would not feel comfortable being involved in it. This layer is connected to the previous one throughout the growing tree (having some roots in this layer) that "*represents growth & expansion, growing in knowledge and learning new things*" (Public contributor 5). Thus, showing the interconnectivity between public involvement principles and those of data science and artificial intelligence.

## Discussion

This participatory evaluation of the co-production process using creative methods has illustrated everyone’s experience of being involved in the project to co-design and co-develop new materials around data science and AI. The evaluation revealed that the entire project was in line with high-quality co-production and public involvement standards: sharing power, up-skilling/learning new things, and providing inclusive and accessible space.

Public involvement requires planning and time [48], and creative approaches (such as photovoice and the mandala) might require additional resources and time but offer a novel way of presenting public contributors' experiences. Photovoice is often seen as an accessible method of participatory approaches; however, a significant challenge is the digital literacy needed to utilize photographic tools (i.e. smart phones, cameras, etc.). This study demonstrated both the accessibility of photovoice, the techniques and adequate support needed to circumvent the usual pitfalls of the common challenges of such a strategy.

The use of nonverbal communication, in this instance, photography and visual mediums, overcomes conventional barriers of language. Photos are known to enable people to make and share their experiences [49]. In this study, we could see how everyone communicated their feelings, thoughts and responses in a way that did not need to be translated. In public involvement, we often work with diverse groups who have mastery of separate languages. Although this is an amazing dynamic, it often means the communication between parties can be difficult to navigate; however, the use of photos and images allowed us to form new ways of communicating that saw past our expected barriers of language. Researchers using photovoice often utilise interview or focus group transcript data to answer research aims rather than photos [41]. Our approach was to put photos at the centre of the analysis and dissemination, hence the creation of the mandala.

The project was tackling a notoriously dense and inaccessible topic—data science and AI. However, this project presented a method of communicating these intense topics and subjects in a manner that was easy to ingest both for everyone. The final application of this methodology was the visualization of a mandala where the independent stories from each of our participants were sewn together into a greater narrative of our research. This method was particularly impactful as it took the place of a traditional findings and critical analysis section, and in its place, we had this visual and deeply emotional reflection on our shared perspectives on this work. Broomfield [50] used creative methods during her public involvement activities and found that discussion based on metaphors of things brought creativity in group members to describe the involvement group they were a part of, understand each other views and thus see different perspectives to identify group dynamics and relationships. This was also our experience when we built the mandala.

The evaluation revealed that their motivations for joining the project were important. These potentially also influenced to the point public contributors’ willingness to sustain their involvement throughout the project. This aligns with previous research with public contributors emphasising that motivation to make a difference is an important aspect of why public members join the research project as public contributors [51]. The public mostly supports clinical AI but has some reservations [4]. Our findings suggest new learnings around AI and data science targeted at researchers and the academic community—it is that extra care and ethical approaches should be taken while deploying data science and AI within the healthcare setting. Meaningful public involvement is required to ensure ethical data science and AI. This could be achieved by following established standards, for example, key principles for public involvement and engagement in data-intensive health research [52].

Public involvement, by its nature, is an ‘alternate’ form of research. The use of creativity allows for the established walls and rules of conventional academia to be broken down and manipulated into a shape where everyone is able to participate. When both researchers and members of the public are encouraged to be curious, imaginative and creative, we develop a holistic and deep understanding of what we are trying to explore. We shed the barriers of a convention that ultimately limit our ability to participate, engage and connect both with each other and the problems we seek to understand.

Creative approaches alongside reflexivity allowed for accessible, inclusive, and therefore more ethical involvement of public partners in the evaluation process around the complex and often abstract topics like data science and AI. Reflexivity offered additional time and space for deeper consideration that is needed for ethical involvement. Following Helen Kara [33] we perceive reflexivity as an opportunity for debiasing, taking a step back to consider what one knows about the process and how one is aware of it. Public contributors can have biases when expressing their views [53]. Thus, our evaluation process showed that reflexivity could address that problem. We embedded public contributors’ reflexivity throughout the evaluation process and when co-writing this paper. The following two section consists of reflections from two public contributors who are co-authors.

### Reshma’s reflections

I had experience as a patient and public contributor in other branches of medicine, but when I started to participate with Imperial College as a public contributor on this project, I was not aware of the existence of the concept of AI. I had absolutely no background in science or mathematics.

I then discovered the prominent role artificial intelligence will play in medicine. I have had caring responsibilities towards my family and decided to understand and engage with this science to help my family and myself when we are unwell and, indeed, be useful and active participants in the health service. I can now help as a patient and public contributor in developing tools and strategies for the dissemination of AI in medicine to others like myself. I am now a part of two steering groups for artificial intelligence in dementia and a PPI member for artificial intelligence in respiratory care in intensive care medicine. I think it is imperative that for the patient and the health service to succeed, people understand, as far as possible and actively participate in using artificial intelligence in medicine.

### Suzanne’s reflections

My introduction to Artificial Intelligence came through being involved in a project whereby an algorithm was developed using NHS data for medical diagnostic use. This present project has furthered my understanding while making me realise how little I know.

What did I enjoy most? Certainly, the creation of photos and paintings to express visually the understanding I had gained and the feelings experienced. I recall surveying my flat to choose objects to photograph or paint and being surprised at which items I selected and why. Focusing on the process was illuminating!

It is so easy to have an experience without spending time reflecting on it. In so doing one fails to recognise much of what one has learned and the impact it has had. Thus, Photovoice has been a very affirmative activity and I will continue using art forms for reflection and expressing emotions.

### Strengths and limitations

Using a participatory approach allowed us to bring people to offer different perspectives and views and ensured the feeling of ownership among all involved, including public contributors. This aligns with the review of the literature on photovoice in health and public health that photovoice could use participatory analysis [41]. However, photovoice could also be used alongside further transcribed interviews or focus groups, thus allowing data triangulation. Due to the project timeline, this was not possible to achieve. Future research could combine conventional and creative methods to produce more nuanced findings [23].

Due to funding restrictions, the evaluation started when the project was already ongoing. We recognise that we managed to capture the ongoing experience of everyone during three months of photovoice, but for previous months, this was mostly reflective and was not apparent in the findings. Teodorowski and colleagues [54], explored the experiences of researchers and public involvement facilitators in involving and engaging seldom-heard communities in big data research; they recognise that there is no one right approach to working with all communities. Our evaluation suggests that researchers can use creative approaches as one of the effective ways of involving members of the public around data science and AI. We recommend that other researchers consider applying participatory evaluation to explore their co-production process. However, we recognise that participatory evaluation can take longer and require more resources than alternatives.

## Conclusions

This paper reports the findings from the participatory evaluation of the co-production process with public contributors. By using creative approaches and embedding reflexivity, the findings suggest that working with public contributors on technical topics that might seem abstract and full of jargon for lay members of the public can be achieved meaningfully for both researchers and public contributors. The findings showed that the co-production process ensured that everyone involved had a true feeling of ownership over the project.

### Supplementary Information

**Additional file 1.** GRIPP2 short form.

## Data Availability

Anonymised data from photovoice is available on request.
