# Supplementary material for: Participatory evaluation of the process of co-producing resources for the public on data science and artificial intelligence
Source: Res Involv Engagem. 2023 Aug 14;9:67. doi: 10.1186/s40900-023-00480-z (PMC10426152; doi:10.1186/s40900-023-00480-z)
Supplement: Supplementary file 1 — Additional file 1. GRIPP2 short form. [file 40900_2023_480_MOESM1_ESM.docx]

| Section and topic | Item | Reported on page No |
| --- | --- | --- |
| 1: Aim | Report the aim of PPI in the study | 3 |
| 2: Methods | Provide a clear description of the methods used for PPI in the study | 3-5 |
| 3: Study results | Outcomes—Report the results of PPI in the study, including both positive and negative outcomes | 6-10 |
| 4: Discussion and conclusions | Outcomes—Comment on the extent to which PPI influenced the study overall. Describe positive and negative effects | 10-11 |
| 5: Reflections/critical perspective | Comment critically on the study, reflecting on the things that went well and those that did not, so others can learn from this experience | 10 -11 |
